# *MTHFR* (rs1801133) and *SERPINE1*/PAI-1 (−675 4G/5G) gene polymorphisms and their association with spontaneous abortion and missed pregnancy in an ethnic Uzbek population: a case–control study

**DOI:** 10.3389/frph.2026.1943185

**Published:** 2026-09-22

**Authors:** Farkhod Akhmedov, Gulnoza Axmadjonova, Nigora Axmadaliyeva, Habibaxon Negmatshayeva, Saxibaxon Kasimova, Nigina Nasimova

**Affiliations:** 1 Department of Obstetrics and Gynecology in Family Medicine, Bukhara State Medical Institute named after Abu Ali Ibn Sino, Bukhara, Uzbekistan; 2 Department of Obstetrics and Gynecology No. 2, Andijan State Medical Institute, Andijan, Uzbekistan; 3 Department of Obstetrics and Gynecology No. 2, Faculty of Pediatrics, Samarkand State Medical University, Samarkand, Uzbekistan

**Keywords:** gene polymorphism, missed pregnancy, *MTHFR*, PAI-1, *SERPINE1*, spontaneous abortion, thrombophilia, Uzbek population

## Abstract

Spontaneous abortion and missed (non-developing) pregnancy are common early reproductive losses in which disturbed folate metabolism and inherited thrombophilia are implicated. Variants of methylenetetrahydrofolate reductase (*MTHFR*) and of the plasminogen activator inhibitor-1 gene (*SERPINE1*/PAI-1) may increase this risk, but data from Central Asian populations are scarce. We assessed the association of *MTHFR* rs1801133 (c.665C>T; p.Ala222Val, classically C677T) and the *SERPINE1*/PAI-1 −675 4G/5G promoter insertion/deletion polymorphism (rs1799889) with early pregnancy loss in a case–control study of ethnic Uzbek women. Eighty-one women with a history of spontaneous abortion (*n* = 40) or missed pregnancy (*n* = 41) and 83 women with a physiologically progressing pregnancy were genotyped. In controls, both loci conformed to Hardy–Weinberg equilibrium. The *MTHFR* Val allele (40.1% vs. 20.5%) and the Val/Val genotype (22.2% vs. 3.6%) were significantly more frequent in cases, the mutant homozygote conferring an approximately sevenfold increase in odds (OR 7.6, 95% CI 2.5–23.3; *p* < 0.01), whereas the Ala/Ala genotype was associated with lower odds of loss (OR 0.4). Similarly, the PAI-1 4G allele (46.9% vs. 30.1%) and the 4G/4G genotype (25.9% vs. 6.0%) were over-represented in cases (4G/4G: OR 5.5, 95% CI 2.1–14.2; *p* < 0.01). Associations were strongest in women with spontaneous abortion. Exploratory ROC analysis indicated moderate, high-specificity discrimination, greatest for the mutant homozygous genotypes (*MTHFR* Val/Val AUC 0.69; PAI-1 4G/4G AUC up to 0.73). In ethnic Uzbek women, the *MTHFR* Val/Val and PAI-1 4G/4G genotypes were associated with spontaneous abortion and missed pregnancy; given the case–control design, these are associations requiring confirmation in larger studies.

## Introduction

Spontaneous abortion is among the most frequent complications of human reproduction. A substantial proportion of conceptions are lost before or around implantation, and an estimated 10%–15% of clinically recognised pregnancies end in miscarriage confirmed by ultrasonographic or histological examination (1). The majority of these clinically recognised losses occur in the first trimester, in which embryonic chromosomal aberrations represent the single most frequent underlying cause (2, 3). Missed, or non-developing, pregnancy is defined as arrest of embryonic or fetal development with retention of the conceptus. It constitutes a distinct but closely related phenotype within the spectrum of early pregnancy loss. Beyond fetal chromosomal factors, maternal constitutional characteristics, in particular those affecting one-carbon (folate) metabolism and the coagulation–fibrinolysis balance, are increasingly recognised as biologically plausible and potentially modifiable contributors. Beyond the folate and haemostatic pathways, maternal gene polymorphisms have also been implicated in other adverse pregnancy outcomes, such as preeclampsia (4), while local genital-tract immune and infectious disturbances further influence reproductive health (5).

The clinical and public-health significance of early pregnancy loss is considerable. Miscarriage imposes a substantial physical and psychological burden on affected women and couples and, when recurrent, entails repeated clinical evaluation and emotional distress. Maternal age and reproductive history strongly modulate the risk of loss (6), yet a large proportion of cases remain unexplained after standard clinical, anatomical and endocrine assessment, underscoring the need for objective, biologically grounded markers capable of identifying women at elevated constitutional risk. This need is especially pressing in Central Asian settings, including Uzbekistan, where the burden of thrombosis-related and chronic non-communicable disease is high and population-specific genetic evidence to guide risk assessment remains limited (7).

Two maternal molecular pathways dominate the mechanistic literature. The first centres on methylenetetrahydrofolate reductase (*MTHFR*), a key enzyme of folate metabolism. The common *MTHFR* c.665C>T variant (rs1801133; p.Ala222Val, classically designated C677T) encodes a thermolabile enzyme with markedly reduced catalytic activity (8). The consequent impairment of homocysteine remethylation elevates plasma homocysteine, promoting endothelial dysfunction and a prothrombotic state that has been linked to placental insufficiency, pregnancy loss and stillbirth (9, 10). Multiple meta-analyses associate the C677T variant with recurrent pregnancy loss, most reproducibly under homozygous and recessive genetic models, although the reported effect sizes vary appreciably between populations (11–13); this heterogeneity is attributable in part to the pronounced geographical and ethnic variation in 677T allele frequency (14). Beyond its homocysteine-mediated effects, *MTHFR* variation has also been associated with reduced embryo viability and an increased incidence of aneuploidy (15). The second pathway centres on plasminogen activator inhibitor-1 (PAI-1), encoded by *SERPINE1*, the principal physiological inhibitor of fibrinolysis. The −675 4G/5G promoter polymorphism (rs1799889) modulates PAI-1 expression, with 4G homozygotes exhibiting the highest circulating concentrations, reduced fibrinolytic capacity and a prothrombotic tendency that favours microthrombosis within the uteroplacental circulation; meta-analytic evidence links this variant to an increased risk of pregnancy loss (16, 17). More recently, studies in neighbouring populations have examined both loci jointly, supporting a combined, multigenic thrombophilic contribution to miscarriage risk (18).

Despite this substantial body of evidence, several gaps remain. First, the reported associations are inconsistent across studies and populations, especially for *MTHFR* C677T, so risk estimates derived in one ethnic group cannot be assumed to transfer to another. Second, allele frequencies at both loci differ markedly between populations (14, 18). Locally generated molecular-genetic data are therefore required, yet such data for Central Asian and specifically ethnic Uzbek women remain scarce. Third, few studies have evaluated the folate-pathway and the fibrinolytic-pathway variant together while separating the clinically meaningful subtypes of early loss (spontaneous abortion versus missed pregnancy), and while quantifying the discriminatory performance of the variants rather than reporting association alone.

The present study addresses these gaps. To our knowledge, it is among the first case–control investigations to characterise both the *MTHFR* rs1801133 (Ala222Val) and *SERPINE1*/PAI-1 −675 4G/5G (rs1799889) polymorphisms in an ethnically homogeneous Uzbek population; to analyse spontaneous abortion and missed pregnancy as separate clinical subgroups; and to assess the discriminatory value of the risk variants by receiver-operating-characteristic (ROC) analysis alongside conventional measures of association. Accordingly, the study aimed to determine the association of these two polymorphisms with spontaneous abortion and missed pregnancy in ethnic Uzbek women, and to evaluate their potential utility as genetic risk markers. We hypothesised that the mutant alleles (Val and 4G) and, most strongly, the mutant homozygous genotypes (Val/Val and 4G/4G) would be associated with an increased risk of loss, whereas the wild-type genotypes (Ala/Ala and 5G/5G) would be associated with relatively lower risk.

## Materials and methods

### Study design and setting

A hospital-based, observational case–control design was adopted. This design was selected because it is an efficient and appropriate approach for evaluating the association between a defined clinical outcome, early pregnancy loss, and pre-existing constitutional (genetic) exposures. It lets affected and unaffected women be compared with respect to allele and genotype frequencies at candidate loci. The study was carried out at the Andijan State Medical Institute, its affiliated Second City Maternity Complex, and the Central Research Laboratory of the Institute (Andijan, Uzbekistan), and combined retrospective and prospective recruitment. It enrolled women of self-reported ethnic Uzbek origin, so as to minimise confounding by population stratification when interpreting genetic associations. Eligible women were recruited consecutively between 2024 and 2026, and genotyping was performed blinded to case–control status.

### Participants and group definitions

A total of 164 pregnant women were enrolled and allocated to two groups. The case group comprised 81 women with a history of early pregnancy loss and was subdivided *a priori* into two clinically distinct subgroups. Group I comprised women whose pregnancy was complicated by spontaneous abortion, defined as spontaneous expulsion of the conceptus (*n* = 40). Group II comprised women with a missed (non-developing) pregnancy, defined as ultrasonographically confirmed arrest of embryonic or fetal development with retention of the conceptus (*n* = 41). The control group comprised 83 pregnant women of similar reproductive age with an ongoing, physiologically progressing pregnancy at the time of enrolment and no history of spontaneous abortion or missed pregnancy. All diagnoses were established on the basis of standardised clinical criteria and confirmed by ultrasonographic examination. Cases presented with early pregnancy loss in the first trimester, ascertained at approximately 12–14 weeks of gestation. To enrich the case group for inherited (constitutional) risk factors, women with recognised non-genetic contributors to pregnancy loss were not eligible as cases, including pregnancies conceived through assisted reproductive technology, multiple pregnancy, uterine anatomical anomalies, confirmed maternal infection, bleeding disorders, hormonal or decompensated endocrine disorders, severe somatic disease, and known parental or fetal chromosomal abnormality.

### Sample size considerations

The study sample consisted of all eligible women recruited consecutively during the study period (2024–2026) at the participating centres; recruitment was therefore based on availability rather than on a fixed target. Given the effect sizes ultimately observed for the mutant homozygous genotypes, the sample was adequate to detect strong associations. As addressed under Methodological considerations, it provided limited power for smaller effects and for the subgroup analyses.

### Ethical approval

The study was approved by the Ethics Committee of the Andijan State Medical Institute (approval no. 78) and was conducted in accordance with the principles of the Declaration of Helsinki. Written informed consent was obtained from every participant before enrolment.

### Blood sampling and DNA extraction

A peripheral venous blood sample was collected from each participant into an EDTA-anticoagulated tube. Genomic DNA was extracted from peripheral-blood leukocytes using a standard extraction procedure, and the concentration and purity of the extracted DNA (A260/A280 ratio) were verified before genotyping to ensure adequate template quality.

### Genotyping and definition of genetic models

Two polymorphisms were analysed: the *MTHFR* rs1801133 variant (c.665C>T; p.Ala222Val, classically designated C677T), giving the wild-type Ala and mutant Val alleles, and the *SERPINE1*/PAI-1 −675 4G/5G promoter insertion/deletion variant (rs1799889; also reported in the literature as rs1799768), giving the 5G and 4G alleles. Genomic DNA was genotyped at both loci by real-time polymerase chain reaction (PCR) using commercial allele-specific reagent kits (DNA-Technology, Moscow, Russia): the “Genetics of Folate Metabolism” kit for *MTHFR* and the “Cardiogenetics: Thrombophilia” kit for *SERPINE1*/PAI-1, each used according to the manufacturer's instructions on a DTprime/DTlite real-time thermocycler. Genotypes were assigned automatically from the FAM and HEX fluorescence channels by the instrument software, and each participant was classified as wild-type homozygous (Ala/Ala; 5G/5G), heterozygous (Ala/Val; 5G/4G), or mutant homozygous (Val/Val; 4G/4G). Each run included the manufacturer's internal amplification controls, and any ambiguous or amplification-negative reaction was repeated.

Associations were evaluated at both the allelic level (mutant vs. wild-type allele) and the genotypic level, with the wild-type and mutant homozygotes compared against the control group, corresponding to the conventional allelic and recessive/homozygous genetic models used in genetic-association research.

### Statistical analysis

Allele and genotype frequencies were determined by direct counting in each group. Conformity of each locus to Hardy–Weinberg equilibrium (HWE) was tested separately in cases and controls by comparing observed genotype frequencies with those expected under equilibrium, using the *χ*² goodness-of-fit test with one degree of freedom; testing HWE in the control group served as an internal check of sample representativeness and genotyping reliability. Between-group differences in allele and genotype distributions were assessed by the *χ*² test, or by Fisher's exact test where any expected cell count was below five (as for the mutant homozygous genotypes); the strength of association was quantified by the odds ratio (OR) with its 95% confidence interval (CI), calculated relative to the control group. Because this is a case–control study, in which incidence cannot be estimated from the sampled data, the relative risk was not computed and only the odds ratio is reported. These analyses were performed for the whole case group and, separately, for Group I (spontaneous abortion) and Group II (missed pregnancy) versus controls, to determine whether the association differed by clinical subtype. The standardised deviation of observed from expected heterozygosity was expressed as D* = (H_o*—*H_e)/H_e. The discriminatory performance of the mutant alleles and genotypes was explored by ROC analysis, reporting the area under the curve (AUC) with the corresponding sensitivity (SE) and specificity (SP), in the whole case group and in each subgroup; because each predictor is a single dichotomous genotype, these AUC values are presented as exploratory descriptors rather than as formal diagnostic performance. A two-sided *p*-value < 0.05 was considered statistically significant, and all analyses were carried out using standard biostatistical software.

### Methodological considerations and limitations

Several methodological features should be considered when interpreting the results. The single-centre, case–control design and the availability-based sample provide efficient association estimates but do not permit inference of temporal or causal sequence, and no formal *a priori* power calculation was performed; statistical power was consequently limited for small effects and for the subgroup analyses, as reflected in the wide confidence intervals around the homozygous-genotype estimates. Associations were assessed with unadjusted *χ*² tests; individual-level baseline characteristics and potential confounders, including maternal age, body-mass index, parity, gravidity, the number of prior miscarriages, smoking, and periconceptional folate supplementation, were not systematically recorded and therefore could not be compared between groups or entered into a multivariable logistic-regression model, so residual confounding cannot be excluded and all estimates should be read as unadjusted. Folate supplementation is of particular relevance here, since it acts directly on the *MTHFR* pathway, and its absence from the dataset is acknowledged as a limitation. In a sensitivity check using Fisher's exact test (appropriate for the small homozygote cell counts), the principal genotype associations (*MTHFR* Val/Val, Fisher *p* < 0.001; PAI-1 4G/4G, Fisher *p* < 0.001) remained significant and survived a conservative Bonferroni correction, whereas the weaker subgroup associations did not consistently survive correction and are therefore reported as exploratory. In addition, control women were enrolled during an ongoing, physiologically progressing pregnancy and were not systematically followed to delivery; a small number could therefore have experienced a later loss, but such non-differential outcome misclassification would tend to bias the observed associations towards the null rather than generate spurious ones. The candidate-gene approach deliberately targeted two well-motivated loci and did not capture other thrombophilia or folate-pathway variants, gene–gene or gene–environment interactions, or fetal karyotype. Intermediate biochemical phenotypes, namely plasma homocysteine and PAI-1 antigen or activity, were not measured. The mechanisms linking genotype to outcome are therefore inferred from the established literature rather than demonstrated within this cohort. Finally, the observed deficit of heterozygotes among cases, together with the deviation from Hardy–Weinberg equilibrium at the *MTHFR* locus in cases, is consistent with disease-associated enrichment of the mutant homozygote but could also, in part, reflect technical genotyping factors such as heterozygote under-calling; independent replication with detailed assay reporting and, ideally, confirmation of a sample subset by direct sequencing is therefore warranted.

## Results

### Study population and Hardy–Weinberg equilibrium

Of the 81 cases, 40 had experienced spontaneous abortion (Group I) and 41 a missed pregnancy (Group II); 83 women with a normal pregnancy served as controls. In controls, genotype distributions conformed to Hardy–Weinberg equilibrium at both loci (*MTHFR*: *χ*² = 0.11, *p* = 0.71; PAI-1: *χ*² = 1.74, *p* = 0.18), supporting sample representativeness and genotyping reliability. The case group deviated from equilibrium at the *MTHFR* locus (*χ*² = 5.26, *p* = 0.03), reflecting an excess of the mutant homozygote among affected women.

### *MTHFR* rs1801133 (Ala222Val)

The *MTHFR* Val allele was almost twice as frequent in cases as in controls (40.1% vs. 20.5%; *χ*² = 15.0, *p* < 0.01; OR 2.6, 95% CI 1.6–4.22). The wild-type Ala/Ala genotype predominated in controls (62.7% vs. 42.0%; OR 0.4, 95% CI 0.23–0.8), consistent with lower odds of loss, and the mutant Val/Val genotype was markedly over-represented in cases (22.2% vs. 3.6%; *χ*² = 12.7, *p* < 0.01; Fisher's exact *p* < 0.001; OR 7.6, 95% CI 2.5–23.26), whereas the heterozygous genotype did not differ significantly between groups (35.8% vs. 33.7%; *p* = 0.79) (Table 1; Figure 1A).

**Table 1:** Allele and genotype frequencies of MTHFR rs1801133 and SERPINE1/PAI-1 −675 4G/5G (rs1799889) in cases and controls.

Allele/genotype	Cases n	Cases %	Ctrl n	Ctrl %	*χ*²	*p*	OR (95% CI)
*MTHFR* rs1801133 (Ala222Val)							
*Ala*	97	59.9	132	79.5	15.0	<0.01	0.4 (0.24–0.62)
*Val*	65	40.1	34	20.5	15.0	<0.01	2.6 (1.6–4.22)
*Ala/Ala*	34	42.0	52	62.7	7.0	<0.01	0.4 (0.23–0.8)
*Ala/Val*	29	35.8	28	33.7	0.1	0.79	1.1 (0.58–2.08)
*Val/Val*	18	22.2	3	3.6	12.7	<0.01	7.6 (2.5–23.26)
*SERPINE1*/PAI-1 rs1799889 (−675 4G/5G)							
*5G*	86	53.1	116	69.9	9.8	<0.01	0.5 (0.31–0.77)
*4G*	76	46.9	50	30.1	9.8	<0.01	2.1 (1.31–3.22)
*5G/5G*	26	32.1	38	45.8	3.2	0.19	0.6 (0.3–1.05)
*5G/4G*	34	42.0	40	48.2	0.6	0.65	0.8 (0.42–1.44)
*4G/4G*	21	25.9	5	6.0	12.2	<0.01	5.5 (2.1–14.17)

Cases *n* = 81; controls *n* = 83. Reference: control group. Odds ratios (OR) with 95% confidence intervals are reported; relative risk is not shown because it is not validly estimable in a case–control design.

**Figure 1:**
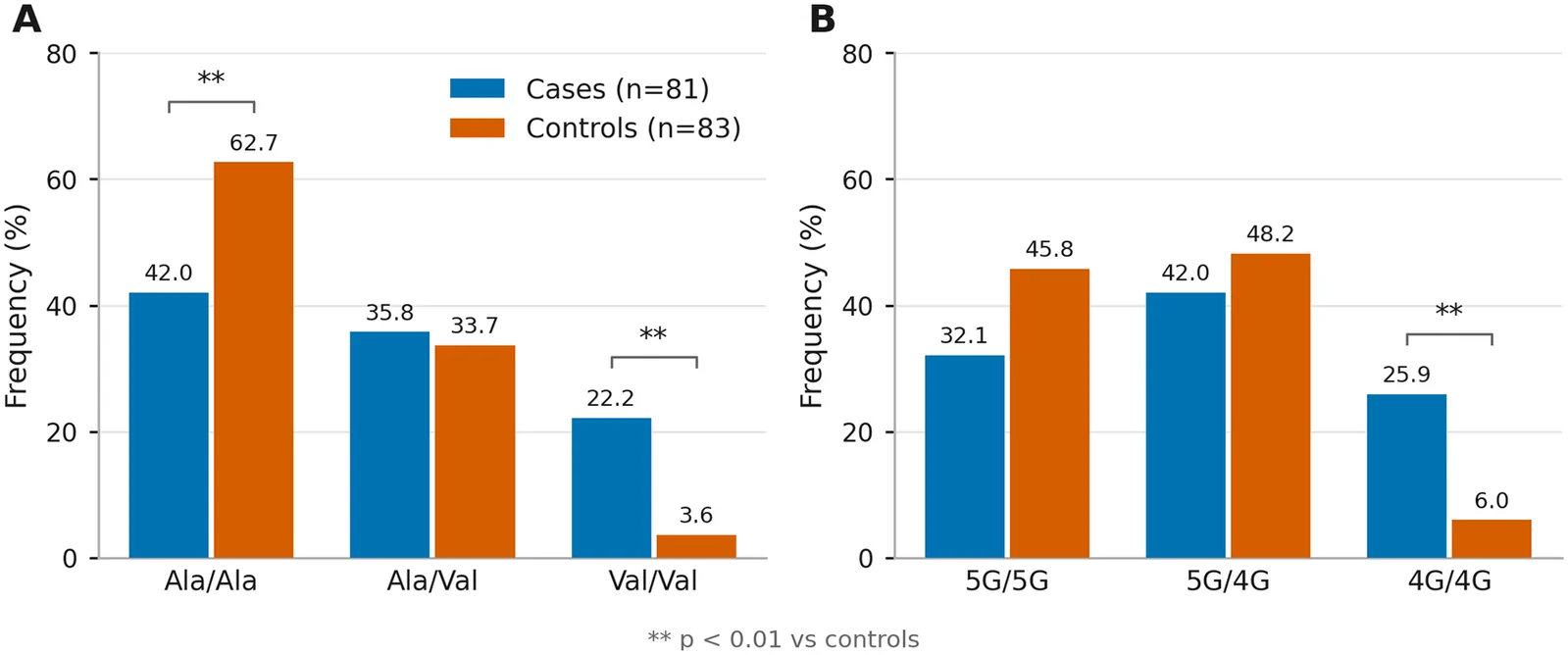
Genotype distributions in cases (*n* = 81) and controls (*n* = 83). **(A)** *MTHFR* rs1801133 (Ala222Val); **(B)** *SERPINE1*/PAI-1 −675 4G/5G (rs1799889).

### *SERPINE1*/PAI-1 −675 4G/5G (rs1799889)

The PAI-1 4G allele was significantly more frequent in cases (46.9% vs. 30.1%; *χ*² = 9.8, *p* < 0.01; OR 2.1, 95% CI 1.31–3.22). The mutant 4G/4G genotype was strongly over-represented in cases (25.9% vs. 6.0%; *χ*² = 12.2, *p* < 0.01; Fisher's exact *p* < 0.001; OR 5.5, 95% CI 2.1–14.17), the wild-type 5G/5G genotype was more common in controls (45.8% vs. 32.1%; OR 0.6, 95% CI 0.3–1.05), and the heterozygous genotype did not differ significantly between groups (42.0% vs. 48.2%; *p* = 0.65). A deficit of heterozygotes was observed in cases (D* = −0.16) and a slight excess in controls (D* = +0.14) (Table 1; Figure 1B).

### Subgroup analysis

Associations were strongest in women with spontaneous abortion (Group I): *MTHFR* Val allele OR 3.0 (95% CI 1.71–5.34) and Val/Val OR 7.7 (95% CI 2.3–26.04); PAI-1 4G allele OR 2.8 (95% CI 1.65–4.88) and 4G/4G OR 6.7 (95% CI 2.38–18.76). In missed pregnancy (Group II) the mutant homozygous genotypes remained significant (*MTHFR* Val/Val OR 7.5; PAI-1 4G/4G OR 4.4), but the PAI-1 4G allele association did not reach significance (OR 1.5, 95% CI 0.85–2.58; *p* = 0.28) (Table 2).

**Table 2:** Risk of mutant alleles and homozygous genotypes in group I (spontaneous abortion) and group II (missed pregnancy) versus controls.

Variant	Subgroup %	Control %	χ²	*p*	OR (95% CI)
Group I—spontaneous abortion (*n* = 40)					
*MTHFR* Val allele	43.8	20.5	14.5	<0.01	3.0 (1.71–5.34)
*MTHFR* Val/Val	22.5	3.6	10.9	<0.01	7.7 (2.3–26.04)
PAI-1 4G allele	55.0	30.1	14.2	<0.01	2.8 (1.65–4.88)
PAI-1 4G/4G	30.0	6.0	13.0	<0.01	6.7 (2.38–18.76)
Group II—missed pregnancy (*n* = 41)					
*MTHFR* Val allele	36.6	20.5	7.4	<0.01	2.2 (1.25–4.0)
*MTHFR* Val/Val	22.0	3.6	10.6	<0.01	7.5 (2.22–25.29)
PAI-1 4G allele	39.0	30.1	2.0	0.28	1.5 (0.85–2.58)
PAI-1 4G/4G	22.0	6.0	7.0	<0.01	4.4 (1.46–13.17)

Reference: control group (*n* = 83).

### Discriminatory performance (exploratory)

Exploratory ROC analysis indicated moderate discrimination (Table 3). The mutant homozygous genotypes performed best, combining high specificity with moderate sensitivity: *MTHFR* Val/Val reached AUC 0.69 (SE 0.72, SP 0.96) and PAI-1 4G/4G reached AUC 0.69 in the whole case group (SE 0.76, SP 0.94), rising to a mean of 0.73 across subgroups. As noted in Methods, these values derive from single dichotomous genotypes and are therefore descriptive rather than a formal evaluation of diagnostic accuracy.

**Table 3:** Exploratory discriminatory performance (ROC analysis) of the risk variants in the case group.

Variant	AUC	Sensitivity	Specificity	OR (95% CI)
*MTHFR* Val allele	0.60	0.80	0.60	2.6 (1.6–4.22)
*MTHFR* Val/Val genotype	0.69	0.72	0.96	7.6 (2.5–23.27)
PAI-1 4G allele	0.69	0.78	0.67	2.1 (1.31–3.22)
PAI-1 4G/4G genotype	0.69	0.76	0.94	5.5 (2.1–14.17)

AUC, area under the ROC curve. Mean AUC across subgroups: 0.64 (*MTHFR* Val), 0.66 (PAI-1 4G), 0.73 (PAI-1 4G/4G). Values derive from single dichotomous genotypes and are exploratory.

## Discussion

In this case–control study of 164 ethnic Uzbek women, two common maternal variants of the folate and fibrinolytic pathways were significantly associated with early pregnancy loss. The mutant homozygous genotypes carried the greatest risk: the *MTHFR* Val/Val genotype increased the odds of loss approximately sevenfold and the PAI-1 4G/4G genotype approximately fivefold, whereas the wild-type genotypes were associated with lower odds and the heterozygous genotypes were not significantly associated, indicating a predominantly recessive pattern of risk. Because controls conformed to Hardy–Weinberg equilibrium at both loci, these estimates rest on an internally valid sample.

The *MTHFR* association is mechanistically coherent. The rs1801133T (Val) allele encodes a thermolabile enzyme whose activity is reduced by up to two thirds in homozygotes (8); diminished enzyme activity impairs homocysteine remethylation, and the resulting hyperhomocysteinaemia drives endothelial injury and a prothrombotic shift linked to placental insufficiency and pregnancy loss (9, 10). The strongly recessive pattern we observed, with risk concentrated in Val/Val homozygotes, fits this dose-dependent enzymatic model.

Our findings are consistent with the broad direction of the meta-analytic literature, including pooled analyses restricted to Asian populations, while also reflecting its well-documented heterogeneity (11, 13). Much of this heterogeneity stems from marked geographical variation in the frequency of the 677T allele (14). In our controls the Val allele frequency was 20.5%, an intermediate value for a Central Asian population, whereas in affected women it reached 40.1%; this near-doubling, with a sixfold excess of Val/Val homozygotes among cases, indicates that the variant is clinically relevant in this population rather than incidental.

The PAI-1 results point to a complementary, haemostatic mechanism: the −675 4G allele is associated with higher plasma PAI-1, reduced fibrinolysis and a prothrombotic tendency that favours microthrombosis in the developing placenta, and updated meta-analyses link the 4G/5G polymorphism to pregnancy loss, most consistently under homozygous models (16, 17). The strong over-representation of the 4G/4G genotype among our cases, its high specificity as a marker, and the observed heterozygote deficit are all consistent with this interpretation. Notably, a recent study in the neighbouring Iranian population likewise implicated both *MTHFR* and PAI-1 variants in miscarriage risk, supporting a shared, regionally relevant genetic contribution (18).

A notable feature of our data is the difference between clinical subtypes: for both genes the associations were strongest in spontaneous abortion, whereas in missed pregnancy the PAI-1 4G allele association at the allelic level lost significance. This dissociation is biologically plausible. An allele acting mainly through placental microthrombosis might exert its clearest effect where a vascular mechanism predominates, as in spontaneous abortion. A missed pregnancy, by contrast, may arise through more heterogeneous mechanisms, including primary embryonic and folate-dependent developmental defects, to which the folate-pathway *MTHFR* variant contributes more uniformly (15). Although the subgroups are small and this interpretation is provisional, it suggests the two variants are not simply interchangeable risk markers.

Considered together, the two variants converge on a single theme: disturbed endothelial function and a prothrombotic uteroplacental environment, reached by a metabolic and a fibrinolytic route. This provides a rationale for the combined, multigenic thrombophilia risk described in recurrent pregnancy loss. From a translational standpoint, the ROC analysis is descriptive rather than evidence of clinical or diagnostic utility, because it rests on single dichotomous genotypes, and it is interpreted with caution: the mutant homozygous genotypes combined high specificity (0.94–0.96) with only moderate sensitivity (AUC 0.69–0.73), a profile at most suited to flagging a higher-risk subset of women who might be prioritised for closer surveillance or evidence-based preventive strategies within current management frameworks (1) than to unselected population screening. Any move towards genotype-guided management would require confirmation in prospective interventional studies, so we frame these implications as hypotheses rather than clinical recommendations.

The study has several strengths, including some of the first molecular–genetic data on these variants in an ethnically homogeneous Uzbek population, Hardy–Weinberg-validated controls, and the simultaneous assessment of two pathway-complementary loci with subgroup and ROC analyses. Its limitations, detailed under Materials and Methods, include the single-centre design and modest sample size, the absence of intermediate biochemical phenotyping, the candidate-gene scope, the lack of adjustment for confounders or multiple testing, and the reliance on unadjusted estimates; the Hardy–Weinberg deviation at the *MTHFR* locus in cases, though expected for a disease-associated genotype, also warrants replication and, ideally, sequencing-based confirmation.

In conclusion, in ethnic Uzbek women the *MTHFR* Val/Val and *SERPINE1*/PAI-1 4G/4G genotypes were associated with spontaneous abortion and missed pregnancy, with the clearest effects in spontaneous abortion. Because this was a case–control study with a limited sample and no adjustment for potential confounders, these results should be interpreted as associations rather than as evidence of causation or of clinical risk prediction, and they require confirmation in larger, prospective studies with full baseline characterisation before any clinical application.

## Data Availability

The datasets generated and analysed during this study are not publicly available because they contain potentially identifying clinical and genetic information about individual participants. The ethics approval obtained for this study and the written informed consent provided by the participants did not cover public deposition of individual-level data. All allele and genotype frequencies supporting the conclusions are presented in full in Tables 1–3 of the article. Anonymised aggregate data are available from the corresponding author (axmedov.farhod@bsmi.uz) on reasonable request.
